# Interactions between β-adrenergic vasodilation and cervical sympathetic nerves are mediated by α_2_-adrenoceptors in the rat masseter muscle

**DOI:** 10.1007/s12576-016-0499-3

**Published:** 2016-11-08

**Authors:** Hisayoshi Ishii, Toshiya Sato

**Affiliations:** Division of Physiology, Department of Oral Biology, School of Dentistry, Health Sciences University of Hokkaido, 1757 Kanazawa, Ishikari-Tobetsu, Hokkaido 061-0293 Japan

**Keywords:** Adrenal nerve, Cervical sympathetic tone, Masseter muscle blood flow, α-Adrenoceptors, Circulating adrenaline

## Abstract

Neural and humoral autonomic mechanisms may be important in the maintenance of blood flow in the masseter muscle (MBF). However, their interactions remain unclear. In this study, we examined interactions between neural and humoral regulation of MBF and investigated the mechanisms mediating these interactions in urethane-anesthetized rats. Stimulation of the adrenal nerve (AN) projecting to the adrenal medulla increased MBF, and this increase was mediated by β-adrenoceptors. Sectioning of the superior cervical sympathetic trunk (CST) significantly inhibited increases in MBF induced by AN stimulation during high activity in the CST, but not during low activity. AN stimulation with clonidine after CST sectioning induced a significant increased in MBF, however phenylephrine had no observable effect. Pretreatment with yohimbine or propranolol significantly inhibited the increase in the MBF. Our results suggest an interaction between β-adrenergic vasodilation evoked by circulating adrenaline and the cervical sympathetic nerves that is mediated by α_2_-adrenoceptors in the masseter muscle.

## Introduction

The autonomic nervous system plays an important role in the regulation of the hemodynamics in jaw muscles, with the autonomic vasomotor responses evoked by neural and humoral mechanisms rapidly and markedly changing the blood flow in jaw muscles [1–3]. Parasympathetic vasodilation in jaw muscles, especially in the masseter muscle, is known to be evoked by a trigeminal [2] or a vagal-mediated reflex [4, 5]. However, sympathetic vasoconstriction is under tonic control from the superior cervical sympathetic trunk (CST) [6]. It has also been reported that the release of circulating adrenaline through activation of the sympathoadrenal system is involved in vasodilation in the masseter muscle through a β-adrenergic mechanism [7]. These observations suggest that neural and humoral autonomic mechanisms and their interactions may be important for the maintenance of blood flow in the masseter muscle (MBF) and the activity of the masseter muscle.

Stress and chronic pain associated with jaw muscle dysfunction, such as fibromyalgia, are known to modulate sympathetic nerve activity, inducing changes in the cardiovascular parameters, such as blood pressure and regional blood flow [8–10]. Sympathoexcitation can cause an increase or decrease in MBF [7, 11, 12]. For example, cold-pressor stimulation has been reported to induce an increase in intramuscular blood volume in the human masseter muscle [11], while acute stress, such as noise, air jets, and noxious cutaneous stimuli, has been found to reduce blood flow in the rabbit masseter muscle [12]. The reasons for these differences in the effects of such stimuli are not clear. However, the interaction between cervical sympathetic nerves (neural) and the sympathoadrenal system (humoral) may be involved in the regulation of MBF during sympathoexcitation. Details of the interaction between these mechanisms in the regulation of MBF are not fully understood.

**Fig. 1:**
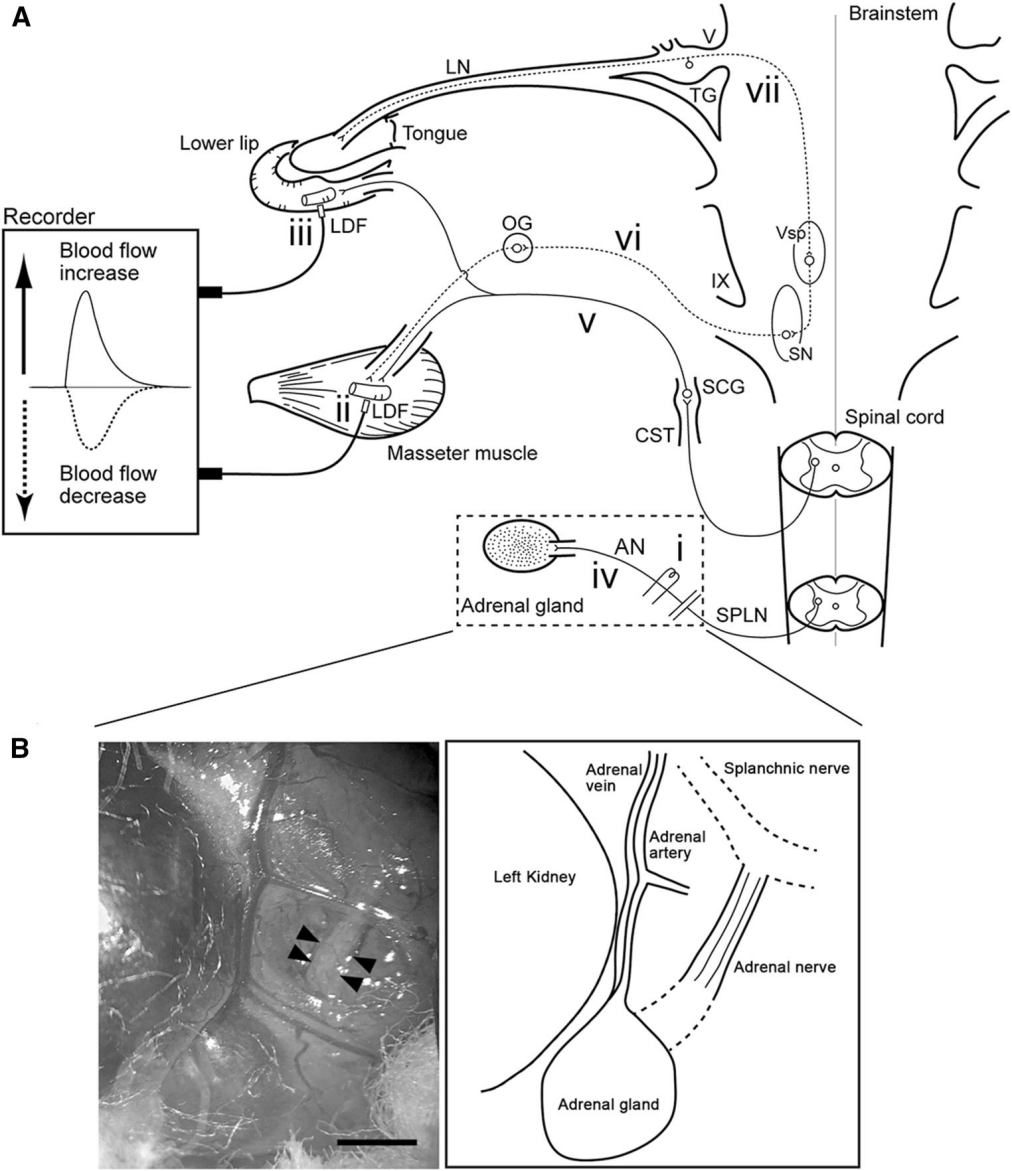
Schematic representation of the sites of electrical stimulation and blood flow measurement in rats. **a** Stimulation site at the peripheral cut end (*i*) of the adrenal nerve (*AN*), which is derived from the splanchnic nerve (*SPLN*). Blood flow was measured at the site of the masseter muscle (*ii*) and lower lip (*iii*) using a laser-Doppler flowmeter (*LDF*). *Solid lines*: *iv* Sympathetic preganglionic neurons projecting to the adrenal medulla through the AN, *v* sympathetic vasoconstrictor fibers to both the masseter muscle and lower lip from the superior cervical ganglion (*SCG*) of the cervical sympathetic trunk (*CST*). *Dashed lines*: *vi* Parasympathetic vasodilator fibers to the masseter muscle and lower lip from the salivatory nuclei (*SN*), *vii* trigeminal sensory inputs to the brainstem. *LN* Lingual nerve, *OG* otic ganglion, *TG* trigeminal ganglion, *Vsp* trigeminal spinal nucleus, *V* trigeminal nerve root, *IX* glossopharyngeal nerve root. **b** Photograph (*left*) and illustration (*right*) of the area indicated by the *dashed rectangle* in **a** showing the left AN to the adrenal gland in a supine rat. *Arrowheads* indicate the approximate site at which nerve was electrically stimulated. *Scale bar* 2 mm Modified from Ishii et al. [7]

In the study reported here, we explored (1) the effects of electrical stimulation of the peripheral cut end of the adrenal nerve (AN) branch from splanchnic nerve projecting into the adrenal medulla on MBF with either the intact or sectioned CST and (2) the underlying mechanisms mediating these responses in deeply urethane-anesthetized, artificially ventilated, and cervically vagotomized rats (Fig. 1).

## Methods

### Preparation of animals

The experiments were performed on 48 adult male Wistar rats between 9 and 16 weeks of age and weighing between 320 and 450 g each. After induction with inhalation anesthesia (isoflurane), urethane (1 g/kg in 1 ml/100 g body weight) was injected subcutaneously into the backs of the animals. One femoral vein was cannulated to allow drug injection, and one femoral artery was cannulated and connected to a Statham pressure transducer to monitor the systemic arterial blood pressure (SABP) and heart rate (HR). The anesthetized animals were intubated, paralyzed by an intravenous (iv) injection of pancuronium bromide (Mioblock; Organon Teknika, Oss, The Netherlands; 0.6 mg/kg initially, supplemented with 0.4 mg/kg every hour or so after testing the level of anesthesia; see following text), and artificially ventilated via a tracheal cannula with a mixture of 50% air and 50% O_2_. The ventilator (model SN-480-7; Shinano, Tokyo, Japan) was set to deliver a tidal volume of 8.5–10 cm^3^/kg at a rate of 20–23 breaths/min, and the end-tidal concentration of CO_2_ was determined by means of an infrared analyzer (Capnomac Ultima; Datex, Helsinki, Finland), as reported previously [2]. Continuous ventilation in this manner has been shown to maintain an end-tidal concentration of CO_2_ of 40–45 mmHg. The changes in the end-tidal CO_2_ following treatment (from 45 to 35 mmHg) were not responsible for the blood flow changes measured by the method used in this study(data not shown). Rectal temperature was maintained at 37–38 °C with the use of a heating pad. Before the injection of additional pancuronium bromide, the depth of anesthesia was determined as adequate by the absence of any flexion response to a noxious stimulus, such as pinching the digit for approximately 2 s. The criterion for the maintenance of an adequate depth of anesthesia following paralysis was defined as the absence of a reflexive elevation of SABP in response to the noxious stimulus. When the depth of anesthesia was considered to be inadequate, additional urethane (intermittent doses of 100 mg/kg, iv) was administered. At the end of the experiment, all rats were killed by an overdose (approximately 100 mg, iv) of pentobarbital sodium. Experimental protocols were reviewed and approved by the Animal Ethics and Research Committee of the University and were conducted in accordance with the Regulations for the Care and Use of Laboratory Animals of the Health Sciences University of Hokkaido (No. 021). All the animals were cared for in accordance with the recommendations in the current National Research Council guide.

### Measurement of the blood flow and cardiovascular parameters

Changes in the blood flow of the bilateral MBF (*n* = 10) and lower lip blood flow (LBF) (*n* = 10) were monitored using a laser-Doppler flowmeter (Fig. 1) (LDF; model FLO-C1; Omegawave, Tokyo, Japan), as described elsewhere [2, 5–7, 13, 14]. The probes were placed against the masseter muscle after making incisions in the cheek and lower lip without exerting pressure on the tissue. The masseter muscle was ascertained by the naked eye. The LDF values obtained in this way represent the blood flow in the superficial vessels of the tissue [15, 16]. The analog output of the equipment did not provide absolute values, but showed relative changes in blood flow [in arbitrary units (a.u.)] (for technical details and an evaluation of the LDF method, see Stern et al. [17]). The SABP was recorded from a femoral catheter via a Statham pressure transducer. All data were collected online with a LabScribe2 data-acquisition system (iWorx Systems, Washington, NH). The HR and systolic, diastolic, and mean SABP were calculated from the SABP signals (*n* = 7). Vascular conductance (VC) was calculated using the following equation:

$${\text{VC (a}} . {\text{u}} . / {\text{mmHg)}} = {\text{blood flow (a}} . {\text{u}} . ) / {\text{SABP (mmHg)}}$$

Changes in the averaged VC were assessed by measuring the maximum value in the responses.

### Electrical stimulation of the AN

The peripheral cut end of the AN (Fig. 1), which regulates catecholamine secretion from the adrenal medulla [18–20], was stimulated electrically using a bipolar silver electrode attached to an electrical stimulator (model SEN-7103; Nihon Kohden, Tokyo, Japan). For this purpose, a midline abdominal incision was made, and the gut was carefully moved aside and covered with sterile gauze moistened with sterile saline (0.9% NaCl). With the use of a cotton swab, a major branch of the AN to the adrenal gland on the left side was cleaned of fat and exposed. Electrical stimulation of the AN was delivered for periods of 20 s with supramaximal voltage (10 V) at 20 Hz using 2-ms pulse durations [7] with either intact or sectioning of the CST in the neck (*n* = 10). In all experiments, the cervical vagi were cut bilaterally at the neck before stimulation, which ruled out the involvement of the visceral inputs through the cervical and abdominal vagus nerves.

### Pharmacological agents

All drugs were dissolved in sterile saline. The agonist drugs were clonidine hydrochloride (*n* = 6; 2–10 μg/ml; Wako, Osaka, Japan) and phenylephrine hydrochloride (*n* = 6; 2–10 μg/ml; KOWA, Tokyo, Japan). The following pharmacological interventions were performed: (1) β-adrenergic blockade using propranolol hydrochloride (*n* = 6–10; 1 mg/ml; AstraZeneca, Osaka, Japan); (2) α_2_-adrenergic blockade using yohimbine hydrochloride (*n* = 6; 0.5 mg/ml; Sigma-Aldrich, St. Louis, MO). These drugs were perfused intravenously for 10 min at a flow rate of 0.1 ml/min using a syringe pump (Model ‘22’ Multisyringe; HARVARD, Holliston, MA). The adrenaline (*n* = 5; 100 ng/kg; DAIICHI SANKYO, Tokyo, Japan) was administered by bolus injections in volumes of 0.01–0.1 ml. The administration of a similar volume of saline had no measurable effect on the cardiovascular parameters (data not shown). The responses evoked by stimulation of the AN after the administration of various drugs were determined at least 5–10 min after the injection when changes in the blood flow and SABP had reached a steady state. The magnitude of the response after the administration of various drugs was compared with the control response recorded before the administration. The effectiveness of the blockade using yohimbine was assessed by the absence of a vasoconstrictor response to clonidine (10 μg/ml) (Fig. 5a). The effectiveness of the blockade using propranolol was assessed by the absence of a vasodilator response to isoproterenol hydrochloride (100 ng/kg; KOWA) (data not shown). The dose of adrenaline chosen for our study was 100 ng/kg, as this dose has been shown to produce a significant increase in MBF [7].

### Reverse transcription-PCR analysis

The urethane-anesthetized rats were killed by cervical dislocation, and the masseter muscles and lower lips were excised and used for subsequent analyses (*n* = 5). The tissue was homogenized in 50 mg/ml of TRIzol/mRNA reagent (Gibco BRL, Grand Island, NY), and the total RNA was extracted as described in the protocol provided by the manufacturer. Total RNA (1 μg) from the tissue was used for the reverse transcription (RT)-PCR analyses in a final volume of 50 μl. The PCR amplifications were performed using specific primers for α-adrenoceptor isoforms (α_1A_, α_2A_, α_2B_, and α_2C_) as described in Table 1 [21]. The size of the expected fragments for each isoform was: α_1A_, 479 (range: 154–632) bp; α_2A_, 265 (1116–1380) bp; α_2B_, 396 (1481–1876) bp; α_2C_, 567 (2031–2597) bp. The RT-PCR was conducted using a one-step RT-PCR kit (Qiagen, Holden, Germany). Template RNA in each tube was reverse transcribed at 50 °C for 30 min, subjected to initial PCR activation at 95 °C for 15 min, and then subjected to PCR cycling (35-step cycle) consisting of denaturation at 94 °C for 1 min, annealing at 58 °C for 1 min, and extension at 72 °C for 1 min. A 10-μl portion of the amplified product was resolved on 2.0% agarose gels (NuSeive 3:1; FMC Products, Rockland, ME) containing 0.01% GelRed™ (Biotium, Fremont, CA), and the DNA was visualized by LED transillumination (Wako, Osaka, Japan). The gel images were captured by a digital camera, recorded on a Macintosh computer, and quantified using ImageJ 1.46r software. The products were normalized with that of the housekeeping gene, glyceraldehyde-3-phosphate dehydrogenase (GAPDH, 452 bp) from the same template and shown as a ratio [22].

**Table 1:** Oligonucleotide sequences of specific primers for rat α-adrenoceptor isoforms (5′→3′)

Isoform	Sense primer	Antisense primer
α_1A_	GTGATCCTCTCAGTGGCCTG	CGACAGTACATAACCAGAAT
α_2A_	CACGTTCGTGCTGGCGGTGGTGAT	GGTCTGTAAGCAGCACAGCCCGAG
α_2B_	CACCTTTGTGCTGGCCGTGGTCAT	CAGCATTTTTGTCCTTTCCCCTTC
α_2C_	CTTCACCTTCGTGTTGGCGGTGGT	GGGAGGGTCTCCTTTCTGGTGCAG

GenBank accession numbers for the above adrenoceptor isoform sequences are as follows: α_1A_: U07126; α_2A_: M62372; α_2B_: M32061; α_2C_: X57659

### Statistical analysis

All numerical data are presented as the mean ± the standard error of the mean. The statistical significance of the observed changes was assessed using the paired Student’s *t* test or analysis of variance (ANOVA) followed by a post hoc test [Fisher’s protected least significant difference (PLSD) test] and a contrast test. Differences were considered to be significant at *P* < 0.05. Data were analyzed using a Macintosh computer with StatView 5.0 (SAS Institute Inc., Cary, NC) and SuperANOVA (ABACUS Concepts, Berkeley, CA).

## Results

### Effects of electrical stimulation of the peripheral cut end of the AN on the hemodynamics in the masseter muscle and lower lip, and the SABP before and after sectioning of the CST

**Fig. 2:**
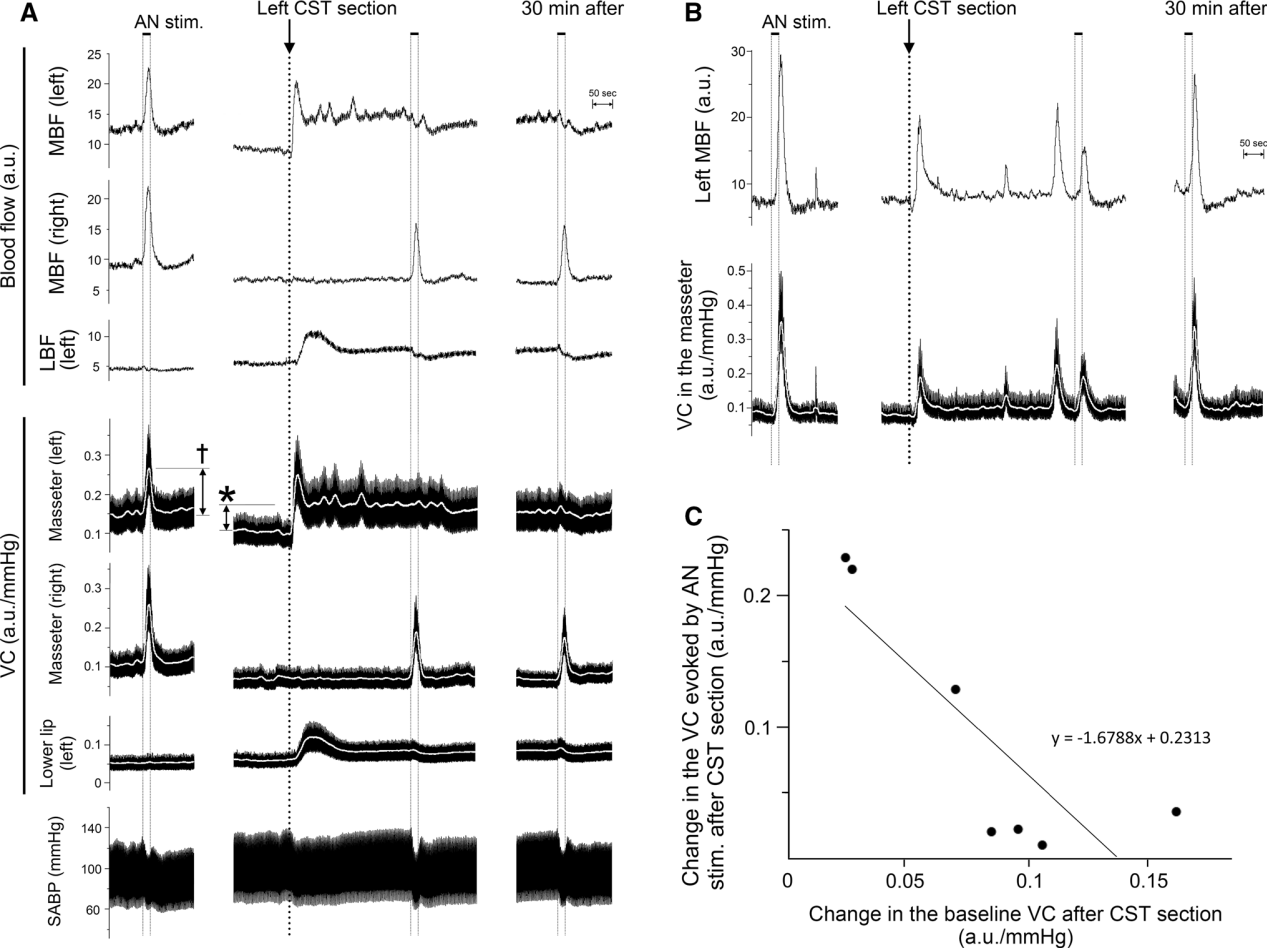
Relationships between cervical sympathetic nerves and the sympathoadrenal system in the regulation of cardiovascular parameters in the rat. **a** Typical examples of the effects of electrical stimulation of the peripheral cut end of the left AN (at *black horizontal bars*) before and after (5–30 min) sectioning of the left CST (at *arrow*) on the blood flow in the masseter muscle (*MBF*) on both sides, lower lip (*LBF*) on the *left side*, vascular conductance (*VC*) of the measurement sites and systemic arterial blood pressure (SABP) during high spontaneous activity in the CST. Increases in the VC were assessed by measuring the height of peak (*†*) during the response. **b** Typical examples of the effects of the left AN stimulation before and after sectioning of the left CST on the left MBF and VC in the masseter muscle during low spontaneous activity in the CST. Electrical stimulation of the AN was delivered for 20 s with a supramaximal voltage (10 V) at 20 Hz using 2-ms pulses. Changes in blood flows (*a.u.* arbitrary units) and VC (a.u./mmHg) in the masseter muscle and lower lip and SABP were measured simultaneously. **c** Plot of changes in the VC evoked by AN stimulation vs. changes in the baseline VC after CST sectioning (*) in the masseter muscle. The *white* traces in **a** and **b** indicate the mean VC at the measuring sites

**Fig. 3:**
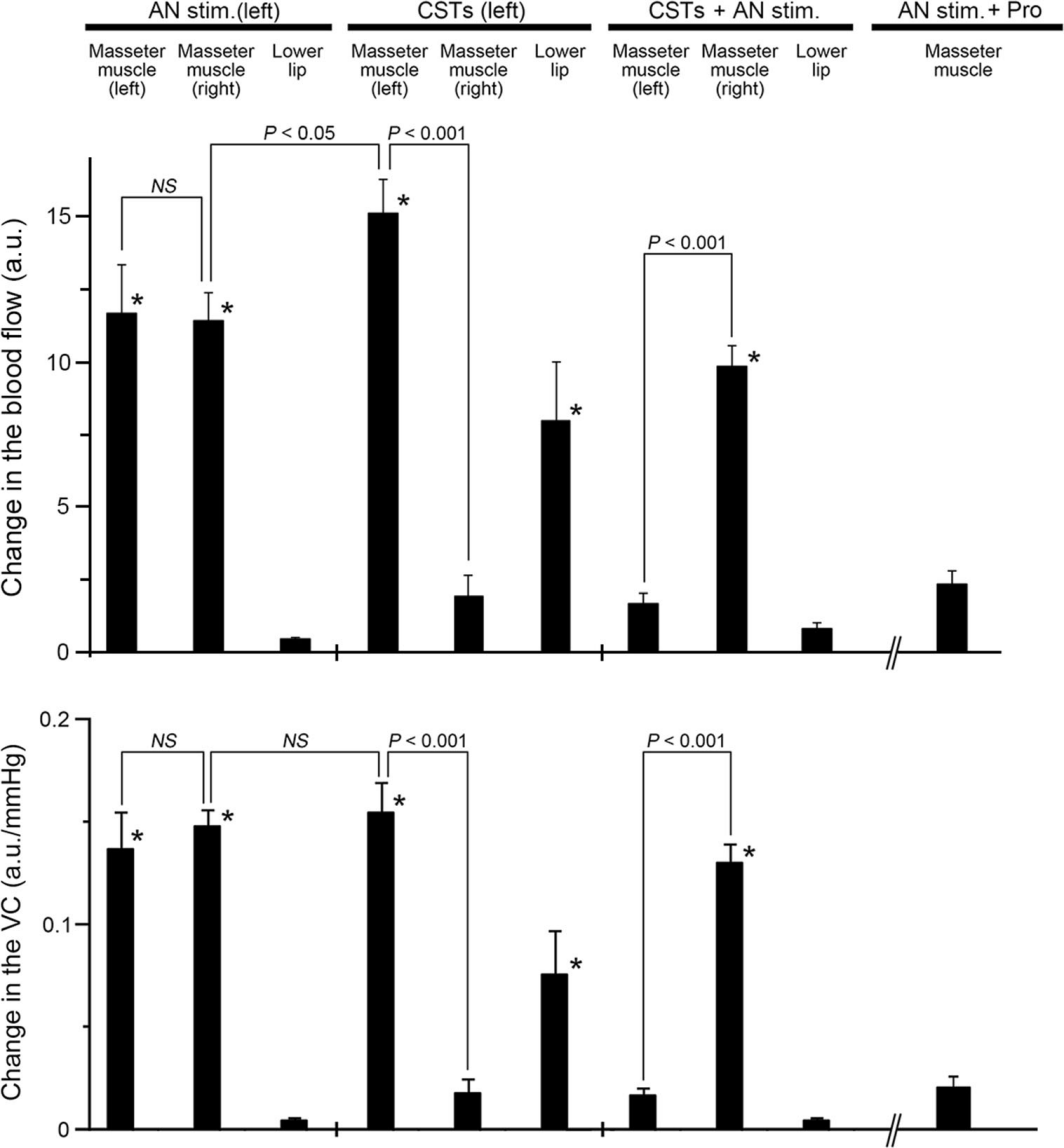
Inhibition by CST sectioning on the increases in the MBF evoked by activation of the sympathoadrenal system during high spontaneous activity in the CST. The mean ± standard error of the mean (SEM) of the changes (*black bars*) in the MBF on both sides, left LBF (*top*), and VC of each measuring site (*bottom*) evoked by electrical stimulation of the left AN (20 s, 10 V, 20 Hz, 2-ms pulses) alone (*AN stim.*), left CST sectioning (*CSTs*), and AN stimulation in combination with CST sectioning (*CSTs + AN stim.*) or propranolol (1 mg/ml; (*AN stim. + Pro*) (*n* = 10 in each group) are shown. Statistical significance of the differences from base value before AN stimulation was determined by analysis of variance (ANOVA) followed by a post hoc test [Fisher’s protected least significant difference (PLSD) test]. *P < 0.001 vs. base value. A significant difference between data sets (*P* < 0.05, *P* < 0.001) or non-significant difference (*NS*) is indicated above the appropriate *square bracket* (ANOVA followed by a contrast test)

Figure 2 shows the effects of electrical stimulation of the left AN on the MBF on both sides, the LBF on the left side, the VC at each of the measuring sites, and SABP before and after sectioning of the left CST. Similar basal levels for MBF and LBF were recorded before the CST was sectioned (Fig. 2a, b). Before CST sectioning, AN stimulation resulted in significant increases in the MBF on both sides, but not in the LBF, and in a slight decrease in SABP (Figs. 2a, 3). Sectioning of the left CST resulted in a significant and a continuous increase in the basal level of the left MBF, but it had no effect on the right MBF (Figs. 2a, 3). The increased level of the MBF is comparable with the spontaneous activity in the CST fibers innervating the masseter vasculature as electrical stimulation of the peripheral cut end of the CST restored the basal level of the MBF to pre-sectioned values, as also demonstrated previously [6]. The increases in the basal level of the MBF were caused by either high or low spontaneous activity (5:2) in the CST (Fig. 2). The increases in MBF evoked by AN stimulation during high spontaneous activity in the CST were not observed at 5 min after ipsilateral CST sectioning, and AN stimulation decreased the MBF at 30 min after CST sectioning (Figs. 2a, 3). The increased in MBF during low spontaneous activity in the CST were evoked by AN stimulation regardless of the presence or absence of sympathetic innervation (Figs. 2b, 3). Changes in VC in the masseter muscle evoked by AN stimulation showed a negative correlation (*r* = −0.83) with changes in the baseline after CST sectioning (Fig. 2c). During the period of high spontaneous activity in the CST, there were significant differences in the left masseter muscle VC before and after the AN stimulation alone or CST sectioning (*P* < 0.001), but not after AN stimulation with CST sectioning (Fig. 3). On the right side, there were significant differences in the masseter muscle VC following AN stimulation regardless of the presence or absence of CST sectioning (*P* < 0.001) (Fig. 3). There was no significant difference between the increase of masseter muscle VC evoked by AN stimulation on the left and right side before CST sectioning (Fig. 3). The increase in the masseter muscle VC evoked by AN stimulation after CST sectioning in the right masseter muscle was significantly larger than that in the left masseter muscle (*P* < 0.001) (Fig. 3). The increases in the MBF and masseter muscle VC evoked by AN stimulation were inhibited significantly by the intravenous administration of propranolol (Fig. 3). The animals exhibited normal systolic and diastolic pressures, mean SABP, and HR during rest. The HR remained unchanged during all of the different treatments (Table 2). There were significant differences in the SABP before and after AN stimulation (*P* < 0.01) (Table 2). The HR at 10 min after the administration of propranolol was 305 ± 9 beats/min. The HR was significantly lower after the administration of propranolol than before its administration (*P* < 0.01). The mean SABP after the administration of propranolol was 117.8 ± 4.6 mmHg. There was no statistically significant difference in the mean SABP before and after propranolol administration.

**Table 2:** Heart rate and systemic arterial blood pressure responses associated with each condition

Heart and blood pressure measures	Baseline	SPLN stimulation	CST section	CST section + SPLN stimulation
HR (beats/min)	433 ± 8	438 ± 8	444 ± 12	438 ± 8
Systolic SABP (mmHg)	141 ± 5	122 ± 6*	134 ± 5	123 ± 8
Diastolic SABP (mmHg)	89 ± 4	75 ± 4*	85 ± 4	77 ± 5
Mean SABP (mmHg)	106 ± 4	91 ± 4*	101 ± 4	92 ± 6

Values in table are given as the mean ± standard error of the mean (SEM)

*SPLN* Splanchnic nerve,* CST* cervical sympathetic trunk,* HR* heart rate,* SABP* systemic arterial blood pressure

* Significant difference from baseline at *P* < 0.01

### Effects of exogenously applied adrenaline on the hemodynamics of the masseter muscle and SABP before and after CST sectioning during high spontaneous activity in the CST

**Fig. 4:**
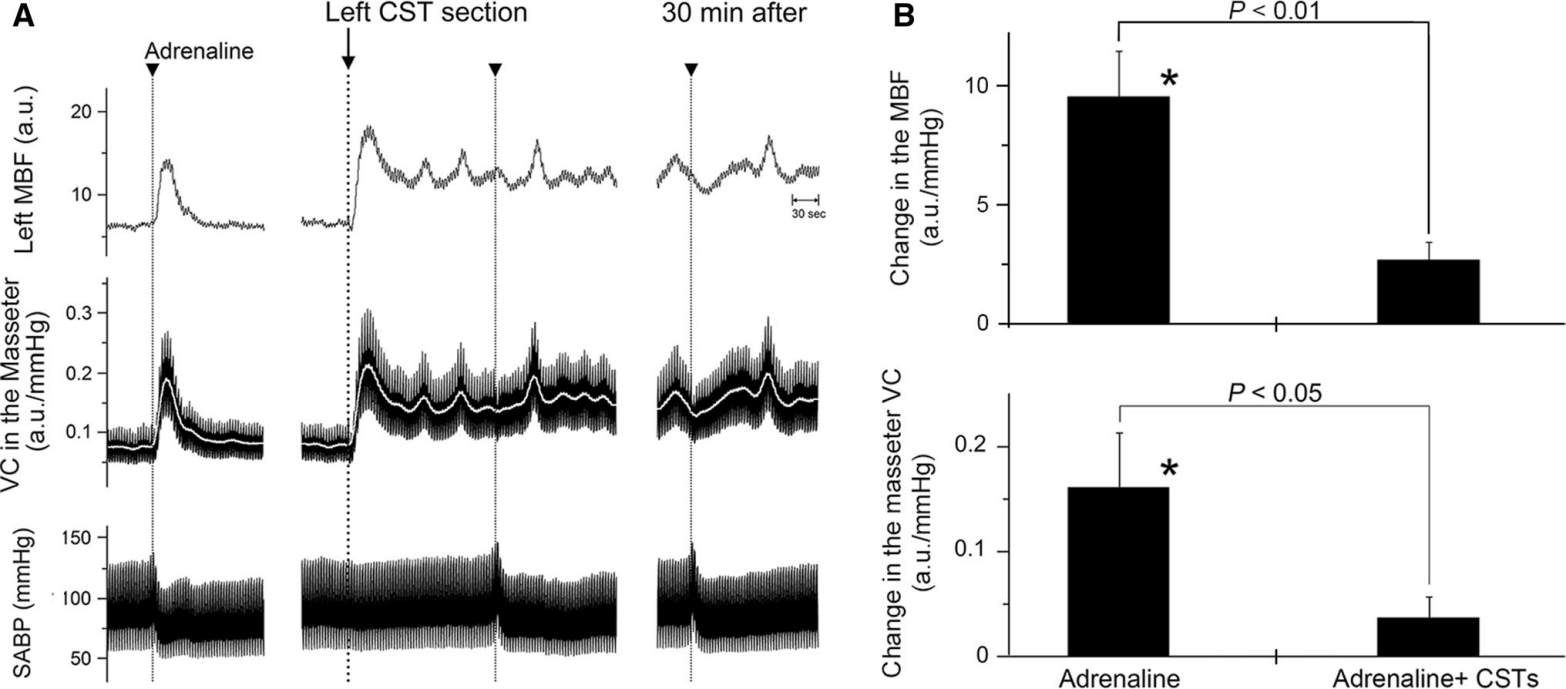
Effects of exogenously applied adrenaline on the hemodynamics of the masseter muscle and on SABP during high spontaneous activity in the CST. **a** Typical examples are shown of the effect of intravenous administration of adrenaline (*arrowheads*, 100 ng/kg) on changes in the MBF and VC in the masseter muscle on the left side, and SABP before and after (5–30 min) the left CST sectioning (at *arrow*). The *white* traces indicate the mean VC for each record. **b** Mean ± SEM of changes (*black bars*) in the left MBF (*top*) and masseter muscle VC (*bottom*) evoked by adrenaline administration alone (*Adrenaline*) and 5 min after adrenaline administration in combination with CST sectioning (*Adrenaline + CSTs*) (*n* = 5 in each group). Statistical significance of the differences from basal values before adrenaline administration were determined by ANOVA followed by a post hoc test (Fisher’s PLSD). *P < 0.05 vs. basal value. Significant differences between data sets (*P* < 0.05, *P* < 0.01) are indicated above the appropriate *square bracket* (ANOVA followed by a contrast test)

Figure 4a shows the effects of the intravenous administration of adrenaline on the MBF and VC in the left masseter muscle and SABP before and after left CST sectioning. Before CST sectioning, adrenaline administration resulted in significant increases in the MBF, accompanied by a slight decrease in SABP (Fig. 4). The MBF increases evoked by adrenaline administration were significantly lower at 5 min after CST sectioning, and adrenaline administration produced a decrease in the MBF that persisted until 30 min after CST sectioning (Fig. 4). There were significant differences in the masseter muscle VC before and after adrenaline administration alone (*P* < 0.05), but its administration after CST sectioning had no significant effect on hemodynamics in the masseter muscle (Fig. 4b). The HR remained unchanged by the administration of adrenaline (425 ± 17 beats/min). Adrenaline administration caused slight decreases in the mean SABP (93.9 ± 5.7 mmHg), but there was no significant difference in the mean SABP before and after adrenaline administration.

### Effects of activation of α-adrenoceptors on hemodynamics in the masseter muscle evoked by AN stimulation and SABP after CST sectioning during high spontaneous activity in the CST

**Fig. 5:**
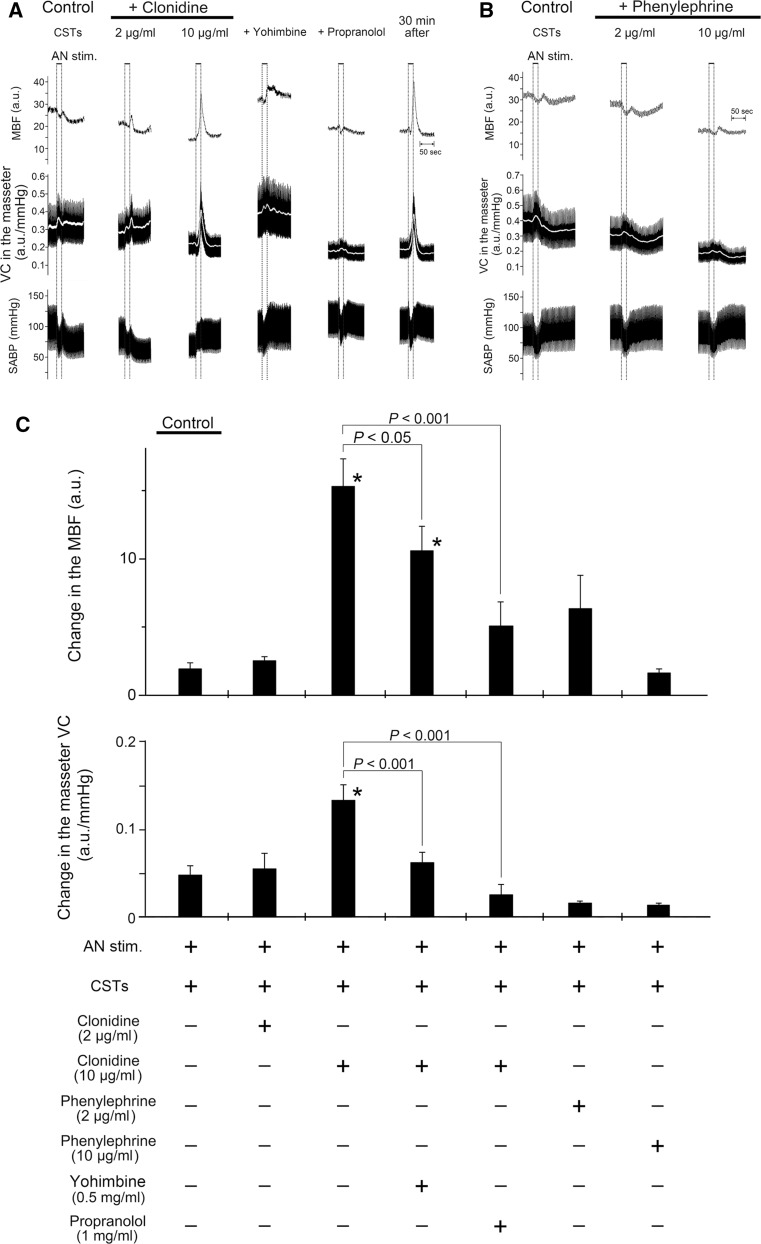
Effects of activation of α-adrenoceptors on changes in the hemodynamics of the masseter muscle and on SABP evoked by AN stimulation after CST sectioning during high spontaneous activity in the CST. **a**, **b** Typical examples of changes in the MBF and VC in the left masseter muscle and SABP evoked by left AN stimulation (*AN stim.*; 20 s, 10 V, 20 Hz, 2-ms) (at *black horizontal bars*) at 10 min after left CST sectioning (*Control CSTs*), in combination with intravenous administration for 10 min (0.1 ml/min) of α_2_-adrenoceptor agonist clonidine (2–10 μg/ml), clonidine (10 μg/ml) and yohimbine (0.5 mg/ml) or propranolol (1 mg/ml) applied together, and 30 min after the injection (**a**), and in combination with administration of α_1_-adrenoceptor agonist phenylephrine (2–10 μg/ml) (**b**). The *white* trace shows the mean VC for the various data. **c** Mean data ± SEM of the changes (*black bars*) in the left MBF (*top*) and masseter muscle VC (*bottom*) evoked by AN stimulation at 10 min after CST sectioning for the various treatments (*n* = 6 in each group). Presence (*+*) or absence (*−*) of treatment is indicated under the *bars*. Statistical significance of the differences from the control before the drug injections determined by ANOVA, followed by a post hoc test (Fisher’s PLSD). *P < 0.001 vs. control. Significant differences between sets of data (*P* < 0.05, *P* < 0.001) are indicated above the appropriate *square bracket* (ANOVA followed by a contrast test)

Figure 5 shows the effects of the intravenous administration of clonidine alone and in combination with yohimbine or propranolol and phenylephrine on the changes in MBF and VC in the left masseter muscle evoked by stimulation of the left AN, as well as changes in the SABP after left CST sectioning. The basal level of the MBF after administration of either clonidine (*P* < 0.01) or phenylephrine (*P* < 0.05) at 10 μg/ml, but not 2 μg/ml, was significantly smaller than that before their administration. The administration of clonidine negated the inhibitory effects of CST sectioning on MBF increases evoked by AN stimulation at 10 min after clonidine injection in a dose-dependent manner (2–10 μg/ml), whereas the administration of phenylephrine did not influence the inhibition (Fig. 5a, b). Increases in MBF evoked by AN stimulation in combination with clonidine (10 μg/ml) after CST sectioning were not observed at 10 min after pretreatment with yohimbine or propranolol, and the response in the MBF returned to near the initial value at 30 min after their administration (Fig. 5a). There was a significant difference in the increases in the masseter muscle VC evoked by AN stimulation before and after 10 μg/ml of clonidine administration (*P* < 0.001) (Fig. 5c). Yohimbine or propranolol pretreatment significantly reduced the increase in the masseter muscle VC (*P* < 0.001) (Fig. 5c). The HR at 10 min after administration of 2 and 10 μg/ml clonidine was 424 ± 21 and 343 ± 5 beats/min, respectively, while that after administration of 2 and 10 μg/ml phenylephrine was 407 ± 7 and 407 ± 8 beats/min, respectively. The mean SABP after administration of 2 and 10 μg/ml clonidine was 88.7 ± 2.5 and 74.6 ± 3.9 mmHg, respectively, while that after the administration of 2 and 10 μg/ml phenylephrine was 127.7 ± 10.9 and 123.5 ± 4.9 mmHg, respectively. There were statistically significant differences in both the HR (*P* < 0.01) and mean SABP (*P* < 0.05) before and after the administration of clonidine at 10 μg/ml.

### Expression of α-adrenoceptor isoforms in the masseter muscle

**Fig. 6:**
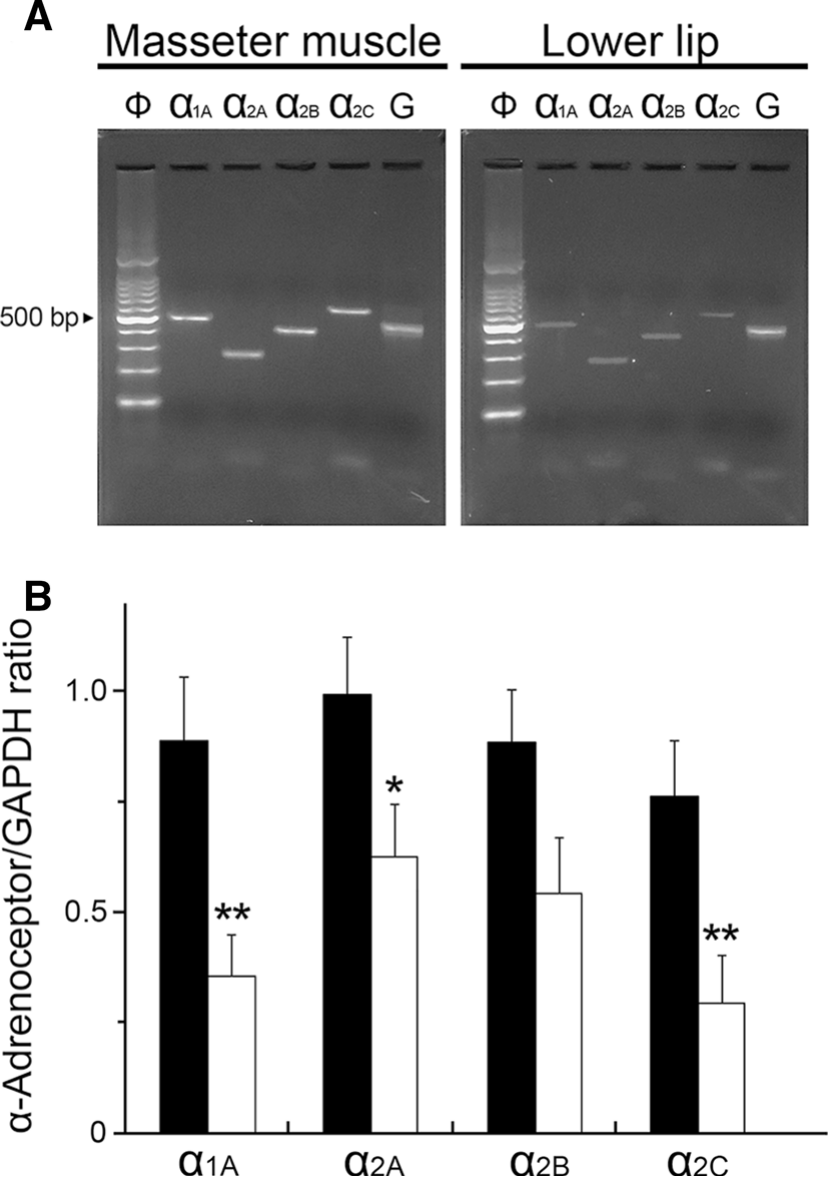
Identification of mRNA expression of α-adrenoceptor isoforms α_1A_, α_2A_, α_2B_, and α_2C_ in the masseter muscle and lower lip using reverse transcription-PCR. **a** Typical examples of the amplified PCR fragments were consistent with the length predicted by the subunit primers. All PCR products were compared with a 100-base DNA ladder (*Φ*).* G* Glyceraldehyde-3-phosphate dehydrogenase (GAPDH). **b** Gene expression of the α-adrenoceptor isoforms in the masseter muscle (*black bars*) and lower lip (*white bars*) normalized with GAPDH from the same template and shown as a ratio (mean ± SEM). Statistical significance of the differences between the masseter muscle and lower lip was determined by ANOVA, followed by a post hoc test (Fisher’s PLSD). *P < 0.05, **P < 0.01 vs. masseter muscle

An RT-PCR was performed to determine which α-adrenoceptor isoforms were expressed in the masseter muscle and lower lip. As shown in Fig. 6, the four isoforms (α_1A_, α_2A_, α_2B_, and α_2C_) were observed at 479, 265, 396, and 567 bp, respectively, as expected (Fig. 6a). The cDNA products of the housekeeping gene GAPDH (452 bp) were expressed in all of the tissue samples. The sequence analyses of these PCR products verified the identity of the various isoforms (data not shown). The relative volumes of their isoforms were significantly higher in the masseter muscle than in lower lip (*P* < 0.05), with the exception of the α_2B_ isoform (Fig. 6b).

## Discussion

Our results show that electrical stimulation of the peripheral cut end of the AN, which induces activation of the sympathoadrenal system, significantly increased the MBF bilaterally in cervically vagotomized rats, while there were no significant increases induced by AN stimulation in the LBF (Figs. 2, 3). The increases in the MBF evoked by AN stimulation were almost completely reversed by the intravenous administration of propranolol (Fig. 3) and not dependent on changes in SABP (Fig. 2). Further, there were no significant differences in the HR before and after AN stimulation (Table 2). These results suggest that the MBF increase elicited by the AN stimulation is not a passive result of any evoked SABP or HR changes and that this increase should be considered to be ‘β-adrenergic vasodilation’. These findings are consistent with the observation that the AN stimulation-induced MBF increase in rats is mediated entirely through the β_2_-adrenoceptors in the masseter muscle [7].

Sectioning of the CST ipsilaterally increased the basal level of MBF, and it significantly inhibited MBF increases induced by AN stimulation during high spontaneous activity in the CST, but not during low activity (Figs. 2, 3). The changes in the MBF evoked by AN stimulation after CST sectioning were similar to those evoked by the intravenous administration of adrenaline (Fig. 4). These results indicate that interactions between the β-adrenergic vasodilation and cervical sympathetic nerves are involved in the maintenance of MBF during high spontaneous activity in the CST. The results of our previous study indicate that AN stimulation elicits MBF increases in cervically sympathectomized rats [7]. The precise reasons for differences in MBF responses are not clear, but anesthesia does influence the mean resting levels of the sympathetic outflow, and differences in MBF responses may be due to differences in the activity of cervical sympathetic nerves depending on the level of anesthesia [23, 24]. Other conditions in our study, such as dose of anesthesia, respiration rate, and SABP levels, are unlikely to account for the discrepancy of MBF response evoked by AN stimulation between our present and previous study [7] because the experimental conditions in both studies are similar.

**Fig. 7:**
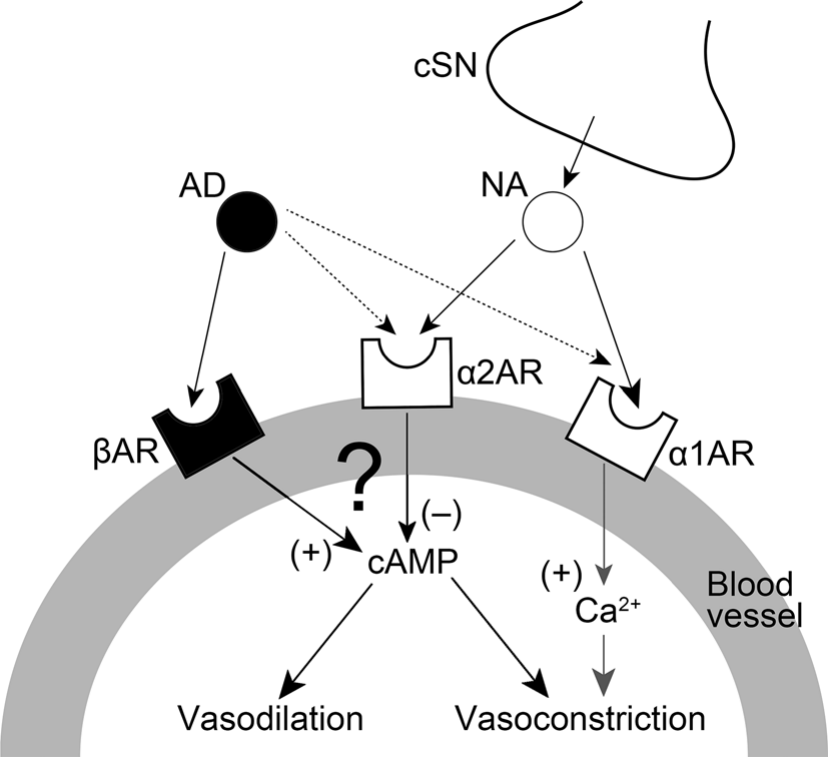
Proposed schema for modulating effects of sympathetic systems in the blood flow in rat masseter muscle. Noradrenaline (*NA*) released by cervical sympathetic nerve (*cSN*) may induce modulation of β-adrenergic vasodilation mediated by circulating adrenaline (*AD*) through the post-junctional α_2_-adrenoceptor (*α2AR*) in the blood vessels in the masseter muscle. *α1AR* α_1_-Adrenoceptor, *βAR* β-adrenoceptor

During high spontaneous activity in the CST, AN stimulation after CST sectioning in combination with the intravenous administration of the α_2_-adrenoceptor agonist clonidine induced a significant increase in the MBF, which was largely suppressed by pretreatment with yohimbine or propranolol (Fig. 5a, c). The administration of the α_1_-adrenoceptor agonist phenylephrine had no effect on the AN stimulation-induced MBF responses after the CST sectioning, while it restored the basal level of the MBF to the pre-sectioned values (Fig. 5b, c). These results suggest that the interaction between the cervical sympathetic nerves (neural) and the sympathoadrenal system (humoral) in the regulation of MBF during high spontaneous activity in the CST is closely related to the α_2_- rather than the α_1_-adrenoceptors via the cervical sympathetic nerves. The α_2_-adrenoceptors are located on sympathetic nerve terminals innervating vascular smooth muscle cells, and they mediate a negative modulation of release of noradrenaline [25, 26]. However, in several blood vessels, post-junctional α_2_-adrenoceptors contribute to the vasoconstriction caused by noradrenaline [25, 27]. The identity of the α_2_-adrenoceptors involved in β-adrenergic vasodilation in the masseter muscle has not been established conclusively; however, the post-junctional α_2_-adrenoceptor must be involved in the response because clonidine administration decreased the MBF consistently in the present study (Fig. 5a). This conclusion is supported by the findings that the three major α_2_-adrenoceptor isoforms (α_2A_, α_2B_, and α_2C_) were expressed in the masseter muscle (Fig. 6). The α_2_-adrenoceptors are coupled predominantly to the inhibitory GTP-binding protein (Gi) which inhibits the activity of adenylyl cyclase [25, 28], while β_2_-adrenoceptors lead to activation of adenylyl cyclase via the stimulatory GTP-binding protein (Gs) [25, 29]. Activation of the β_2_-adrenoceptor has been reported to depend on the basal adenylyl cyclase activity [29, 30], suggesting that adenylyl cyclase-based cross-talk between these receptors may be related to the modulation of β-adrenergic vasodilation in the masseter muscle (Fig. 7).

The β-adrenergic vasodilation is generally accepted to be involved in the modulation of hemodynamics in skeletal muscles during sympathoexcitation [31–34], suggesting an important role in the maintenance of the blood flow and activity of muscles. In the masseter muscle, sympathoexcitation with cold-pressor stimulation induces an increase in intramuscular blood volume in the human masseter muscle, and this increase is reduced significantly by propranolol treatment [11, 35]. These observations suggest that β-adrenergic vasodilation is involved in MBF increases during sympathoexcitation. The functional implications of α_2_-adrenoceptors in the masseter muscle are unclear. However, α_2_-adrenoceptors have been shown to be relatively more prevalent on distal terminal arterioles and affected by local metabolic influences, while larger arteriole constriction has been found to be mediated predominantly by α_1_-adrenoceptors and to be less affected by local tissue factors [36–38]. These observations suggest that an interaction between β-adrenergic vasodilation evoked by circulating adrenaline and cervical sympathetic nerves mediated by α_2_-adrenoceptors would be important for facilitating blood flow and oxygen delivery in the masseter muscle during its exercise.

In summary, our results indicate that cervical sympathetic nerves are involved in β-adrenergic vasodilation in the masseter muscle evoked by circulating adrenaline released from the adrenal medulla during high spontaneous activity in the CST. They thereby suggest that the activation of α_2_- rather than α_1_-adrenoceptors via cervical sympathetic nerves contributes to the observed vasodilation in the masseter muscle. Further studies of the precise mechanisms for the interaction between α- and β-adrenoceptors and their molecular properties involved in the hemodynamics of the jaw muscles should provide a better understanding of the physiological role of neural and humoral regulations of the blood flow in the orofacial area.
