# Correction to: Interactions between β-adrenergic vasodilation and cervical sympathetic nerves are mediated by α_2_-adrenoceptors in the rat masseter muscle

**DOI:** 10.1007/s12576-018-0609-5

**Published:** 2018-04-20

**Authors:** Hisayoshi Ishii, Toshiya Sato

**Affiliations:** Division of Physiology, Department of Oral Biology, School of Dentistry, Health Sciences University of Hokkaido, 1757 Kanazawa, Ishikari-Tobetsu, Hokkaido 061-0293 Japan

## Correction to: J Physiol Sci (2017) 67:699–709 10.1007/s12576-016-0499-3

The article Interactions between β-adrenergic vasodilation and cervical sympathetic nerves are mediated by α_2_-adrenoceptors in the rat masseter muscle, written by Hisayoshi Ishii and Toshiya Sato, was originally published Online First without open access. After publication in volume 67, issue 6, page 699–709 the author decided to opt for Open Choice and to make the article an open access publication. Therefore, the copyright of the article has been changed to © The Author(s) 2018 and the article is forthwith distributed under the terms of the Creative Commons Attribution 4.0 International License (http://creativecommons.org/licenses/by/4.0/), which permits use, duplication, adaptation, distribution and reproduction in any medium or format, as long as you give appropriate credit to the original author(s) and the source, provide a link to the Creative Commons license and indicate if changes were made.

The original article was corrected.

